# Impact of Current Pulsation on BLDC Motor Parameters

**DOI:** 10.3390/s21020587

**Published:** 2021-01-15

**Authors:** Andrzej Sikora, Marcin Woźniak

**Affiliations:** 1 Faculty of Electrical Engineering, Silesian University of Technology, 44-100 Gliwice, Poland; andrzej.sikora@polsl.pl; 2 Faculty of Applied Mathematics, Silesian University of Technology, 44-100 Gliwice, Poland

**Keywords:** BLDC, voltage waveform, ripple current, power losses

## Abstract

BrushLess Direct-Current (BLDC) motors are characterized by high efficiency and reliability due to the fact that the BLDC motor does not require power to the rotor. The rotor of the BLDC motor consists of permanent magnets. When examining the waveform of the current supplied to the motor windings, significant current ripple was observed within one power cycle, where the optimum value would be the constant value of this current during one power cycle. The variability of this current in one motor supply cycle results from the variability of the electromotive force induced in the motor winding. The paper presents a diagram of the power supply system consisting of an electronic commutator and a DC/DC converter made by the authors, and a proposed modification of the power supply system reducing the current pulsation of the motor windings and thus the possibility of reducing energy losses in the motor windings. The paper presents numerous results of measurements which showed a significant reduction in energy losses in the case of low-load operation.

## 1. Introduction

Rare earth magnets allowed for the construction of more efficient and reliable electric motors [1,2,3]. Magnets in modern electric motors are used to create the excitation flux [4]. Classic motors (earlier motor designs) used an excitation winding to generate the magnetic flux, which required a DC supply [5]. Powering the excitation winding (used in earlier designs) generated electricity losses resulting from the flow of the excitation current of the electric machine [6,7]. The loss of electrical energy causes the excitation winding to heat up, thus increasing the temperature of the electrical machine [8]. The elimination of the excitation winding, which causes electric energy losses due to the excitation current flow, made it possible, thanks to the use of modern magnets, to reduce the size of the electric machine while maintaining the same power, or to make the electric machine of the same dimensions with greater power [9,10]. Permanent magnets can be placed on the stator of the electric machine, then it is necessary to use a mechanical commutator as in classic DC machines, such solutions are used, for example, in automotive starters excited by permanent magnets [11,12]. Another solution is to place permanent magnets on the rotor of the machine [13,14]. This solution eliminates the mechanical commutator, which causes frequent failures of DC machines [15,16]. Electric machines with permanent magnets placed on the rotor are divided into two types:

- brushless DC electric motor (Brushless DC) (BLDC) and
- permanent magnet synchronous motor (pmsm).

The article presents issues related to the BLDC [17] motor [18]. A characteristic feature of BLDC brushless motors is the pulsation of the current flowing through the windings (during one cycle) [19,20,21]. The flow of ripple current through the motor windings causes higher power losses in the windings than the current of the same average value and the trapezoidal waveform [21,22]. Considering the fact that drives with BLDC motors are among the most efficient, an attempt was made to limit the losses related to ripple of current flowing in the windings (during one power cycle) [23,24]. The aim of the research is to analyze the current waveforms in a BLDC motor running at idle speed and with a load of various sizes [25,26]. The task of the analysis is to determine the losses resulting from the current flow through the motor windings and to propose a way to minimize them [27,28]. The PMSg80-6B engine Figure 4b powered by an electronic commutator was selected for the tests, in which signals from the hall effect sensors [29] are used to determine the position of the motor shaft.

The paper presents research on optimal model of supplying a BLDC motor to allows for the maximum reduction of losses. For this purpose, two variants of engine power are presented and examined. For both power supply variants, tests were carried out to show that the proposed power supply can efficiently stabilize the system during one power cycle and also reduce energy losses in the motor windings. The tests were carried out for various loads and different rotational speeds of the motor. The main contributions of our work to the research field are electronic commutator circuit designed and made by the authors together with research results on its optimization. The work also includes schematic diagrams of basic power supply elements, as well as designs of printed circuit boards (PCBs) showing the layout of tracks and the arrangement of elements on printed boards. Our main novelties of the paper, proposed from our research finding, is an optimized energy-saving power supply model.

## 2. Description of the Research Methodology

**Figure 1:**
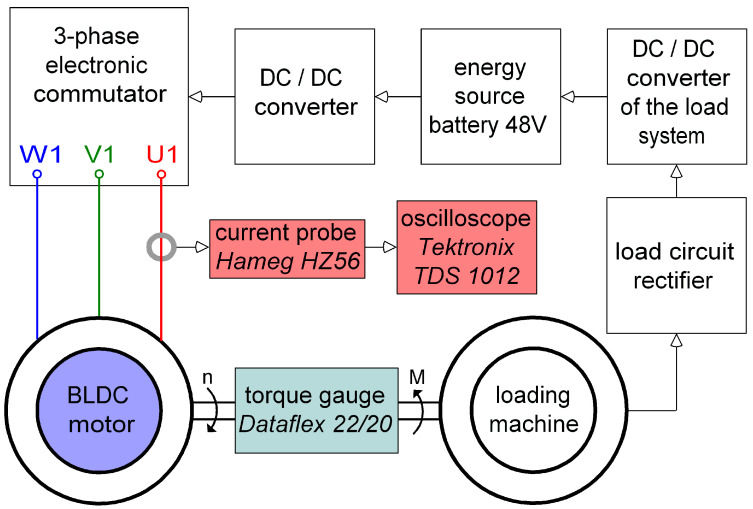
Schematic diagram of the measuring system including the measuring equipment and power supply elements.

It was decided to test the designed and manufactured BLDC motor power supply on a laboratory stand that allows for setting the load torque. The schematic diagram of the measuring stand is presented in Figure 1.

**Figure 2:**
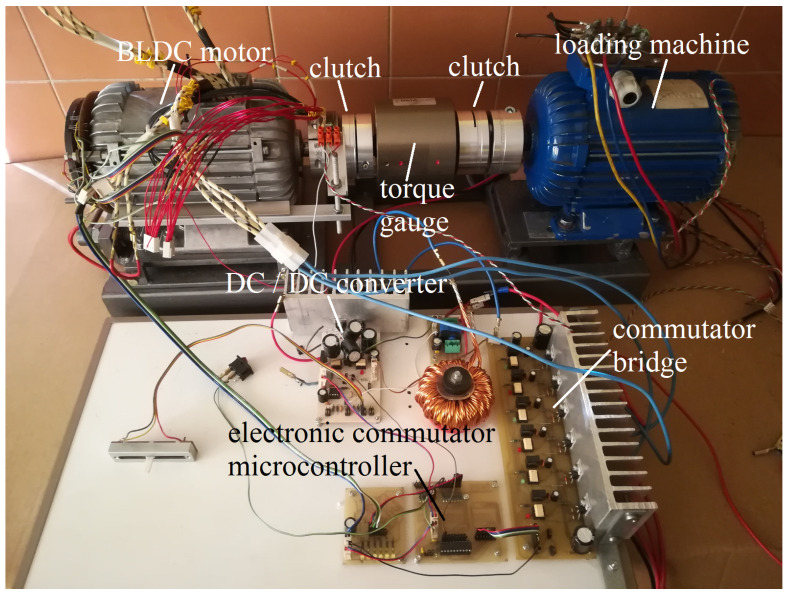
Photography of the test stand model.

The measuring stand was constructed in such a way that the system works in the reverse work mode. This means that the energy given off by the loading machine was used to charge the battery supplying the tested engine. This was achieved thanks to the use of a rectifier and a load converter (DC/DC converter of the load system). As a result, only the loss energy of the tested system was used. A Dataflex 22/20 torque gauge was used to measure the load torque of the engine. The motor supply current was recorded with a Hameg HZ56 current probe connected to a Tektronix TDS 1012 oscilloscope. The test stand was equipped with the tested motor with an electronic commutator and a DC/DC converter allowing for the adjustment of the rotational speed, and a loading machine connected to the shaft of the tested motors through a torque meter. The test stand is presented in the photo in Figure 2.

**Figure 3:**
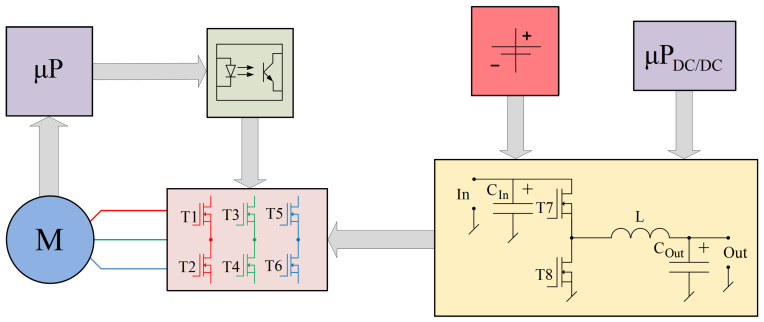
Diagram of the drive system consisting of a microprocessor, optocouplers, an electronic commutator, a permanent magnet motor, a power source, and a two-transistor DC/DC converter with its control microprocessor.

The research was conducted in two stages:

- First, tests were carried out for a system in which the engine was powered classically by means of the system shown in Figure 3, the tests were performed for idling and a few set loads, marked, respectively, M0=0<M1<M2<M3.
- Then, similar experiments were carried out for the system, in which the power supply system was modified to stabilize the current flowing in the motor windings (the implementation of this system will be described later).

At each stage of the research, measurements were made for both directions of rotation. During each stage of the experiment, the data were recorded with an oscilloscope and then analyzed. The analysis was carried out within one engine feed cycle for the pulse with negative values and for the pulse with positive values.

## 3. BLDC Motor and Its Power Supply

A BrushLess Direct-Current (BLDC) motor is a motor in which the coils are stationary and the magnets are on the rotor of the motor. The electric commutator replaced the mechanical commutator with brushes. The design of the motor with permanent magnets placed in the rotor of the motor does not require electricity to be supplied to the rotor; therefore, the BLDC motor does not require the use of a brush system and a classic commutator. The motor is powered by an electronic commutator. The rotational speed is regulated by changing the average value of the voltage supplying the motor. Powering a DC motor with permanent magnets built on the rotor requires sequential switching of voltage to individual windings of the motor. These engines are characterized by the highest efficiency among currently available engines. Due to the lack of brushes, they are also more reliable devices. They are also easier to remove the heat that is emitted in the engine body.

**Figure 4:**
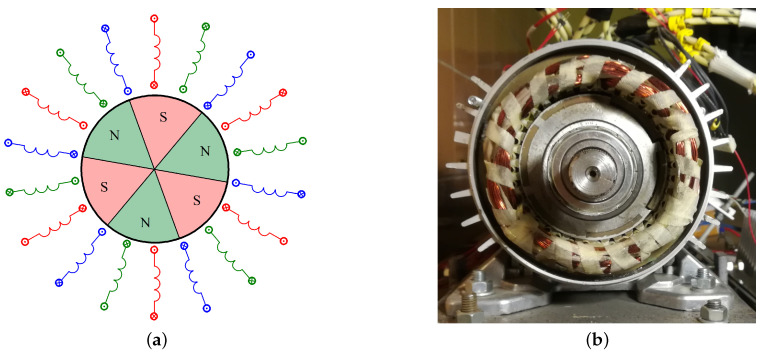
Schematic diagram of a BLDC motor with 3 pairs of magnetic poles in the rotor (**a**) and practical design of motor PMSg80-6B (**b**).

The sequence of powering the motor excited with permanent magnets is strictly determined by the position of the rotor’s magnetic axis in relation to the axis of the stator winding bands. For this reason, it is necessary to determine the angular position of the rotor while the motor is running. Controlling the BLDC motor does not require continuous tracking of the rotor position, it is enough for the rotor angle measuring system to give a signal when the voltage should be applied to a given winding band. Encoders can be used for this purpose. The encoder is a precise meter determining the angular position of the rotor, but it increases the cost of the drive, while transmitting a significant excess of information that is not used for the BLDC motor control [30]. Encoders are not part of the article. Hall sensors can be used to control the BLDC motor to determine the rotor position. In the case of a six-pole motor, the diagram of which is shown in Figure 4a, there are 36 states per one shaft revolution (three full control sequences).

**Figure 5:**
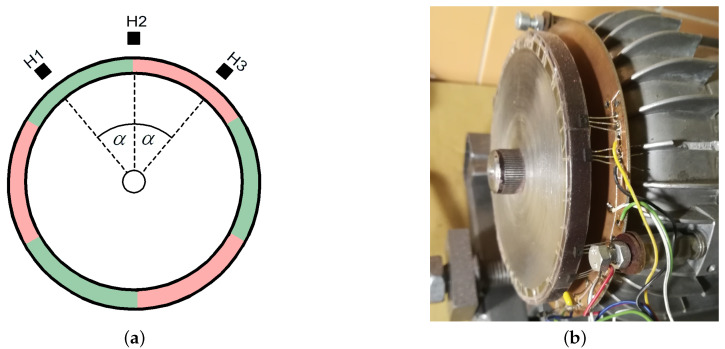
A schematic diagram of the motor shaft position sensor (**a**) and practical application model (**b**).

The sensor in Figure 5 was made with such an angular arrangement of the Hall effect sensors to determine the rotor position necessary to change the control sequence. Hall effect sensors were placed on the outer disc so that they were in the magnetic field of a six-pole disc with glued permanent magnets mechanically coupled to the motor shaft. The external magnetic transmitter was made in such a way that the gap between the magnets was minimal.

**Figure 6:**
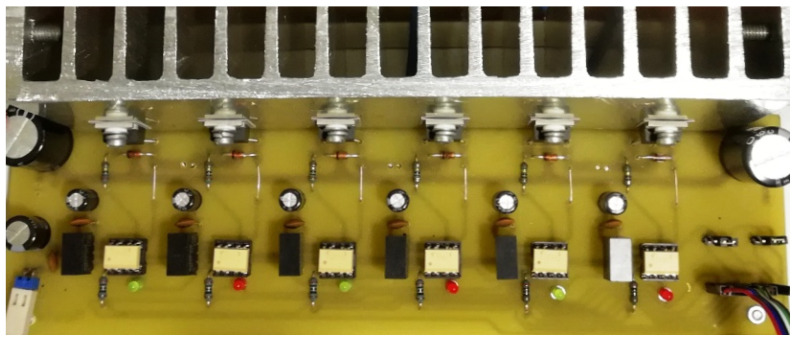
Photo of the bridge supplying the BLDC motor.

**Figure 7:**
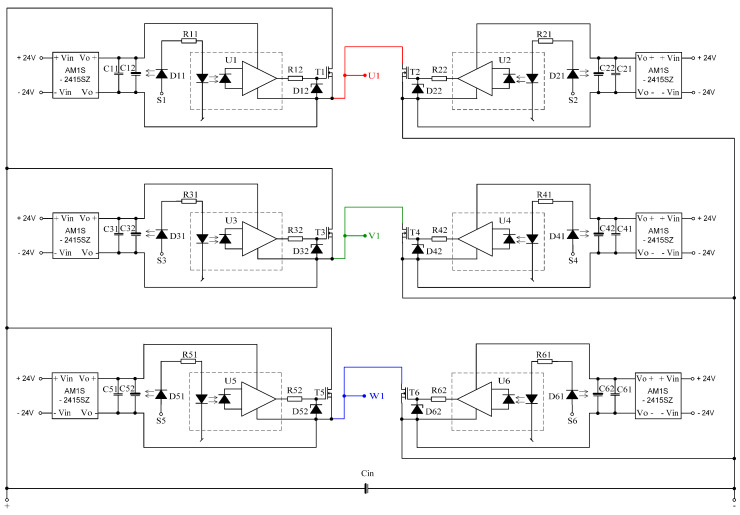
Schematic diagram of the bridge supplying the BrushLess Direct-Current (BLDC) motor.

**Figure 8:**
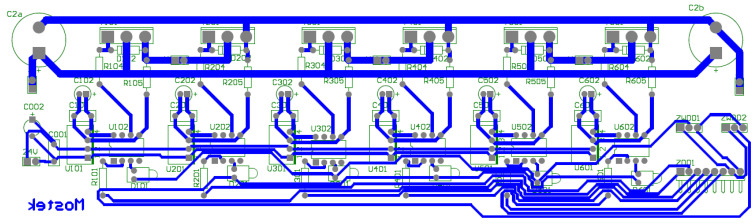
Printed circuit board (PCB) design of the bridge supplying the BLDC motor.

The operation of the brushless motor is based on a given sequence of voltages supplying individual windings. This sequence for a given direction of rotation clearly depends on the angular position of the rotor. The control system of the windings of the three-phase BLDC motor is powered by a system of six transistors operating in a three-phase bridge system. Transistors working as power electronic keys are controlled by a microprocessor system. Control is based on signals from three hall sensors. These sensors were arranged in such a way as to use their signals to determine the position of the rotor, which corresponds to one of the six power sequences. The system of transistors together with the control system is commonly called an electronic commutator. The schematic diagram of the electronic commutator bridge made by the authors is shown in Figure 6, while the photo of the electronic commutator is shown in the Figure 7. Additionally, Figure 8 shows the PCB design on the basis of which the tested electronic commutator bridge was made.

The BLDC motor supply voltage waveform is a trapezoidal waveform. The voltage induced in the windings, coming from permanent magnets, deviates from the shape of the supply voltage waveform, so that the supply current contains a significant ripple component. In the case of idling operation, the instantaneous value of the voltage induced in the windings (during one power cycle) exceeds the value of the supply voltage, causing the instantaneous current flow from the motor to the power source. Powering the BLDC motor with ripple current in its windings produces higher power losses than in the case of powering it with a constant value, equal to the average ripple current (during one power cycle).

**Figure 9:**
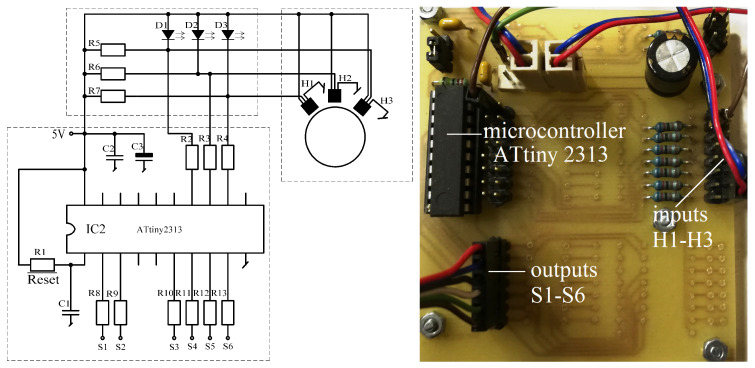
Schematic diagram and photo of the electronic part of the commutator.

**Figure 10:**
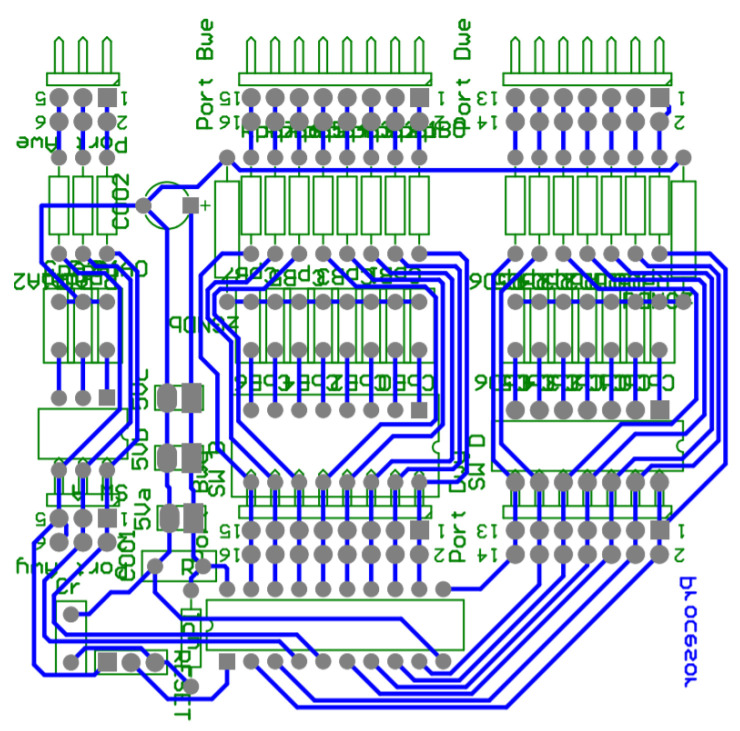
Electronic commutator PCB design.

The electronic commutator system used to power the BLDC motor basically consists of the power electronics and the electronic part (Figure 9) responsible for driving the power transistors based on the indications of the shaft position sensor. In addition, Figure 10 shows the design of the printed circuit board of the PCB system realized during the research.

**Figure 11:**
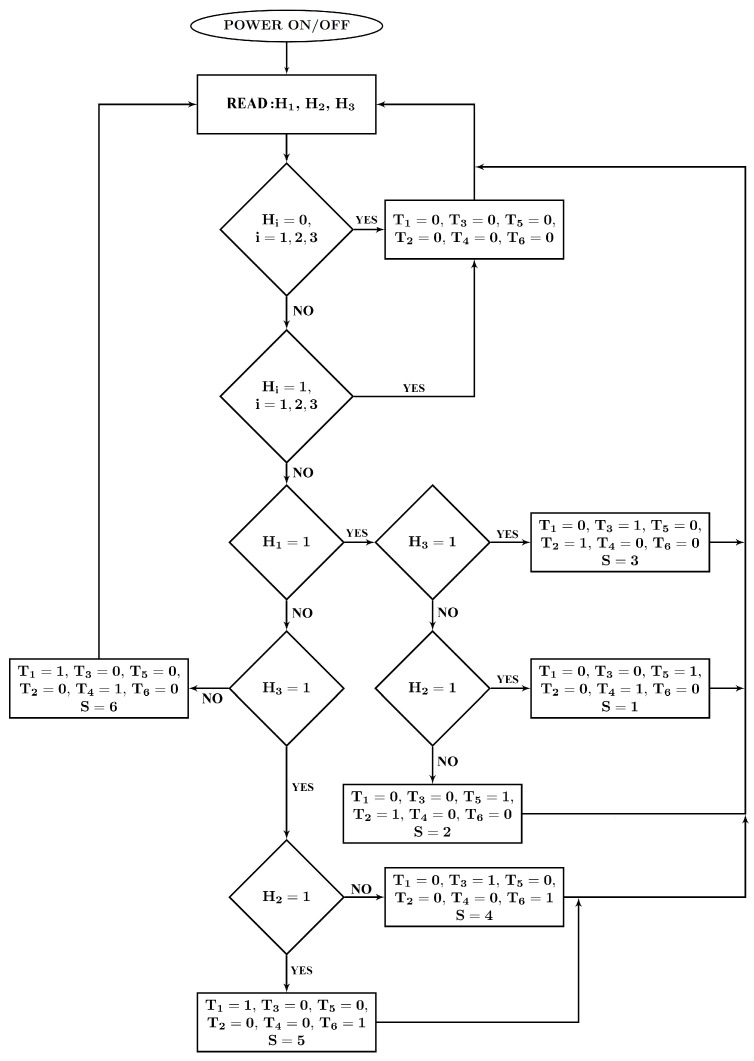
Block diagram of the microcontroller program controlling the power supply of the bridge, where $H_{1}$, $H_{2}$, $H_{3}$ is the input signal from Halls, $T_{1},\ldots ,T_{6}$ state of the transistors, and where 1 means open and 0 closed. The *S* variable represents one of the six possible states of the engine control sequence.

The control unit is the ATtiny 2313 microcontroller, which reads signals from a system of hall sensors cooperating with a magnetic target with the same number of pole pairs as the motor rotor. Depending on the signals supplied from the system of three hall sensors, the microcontroller works out the appropriate sequence of power transistors of the IRF3205 (T1–T6) type with rated parameters 55V and 80A (Figure 7). Figure 11 microcontroller implementation diagram.

**Figure 12:**
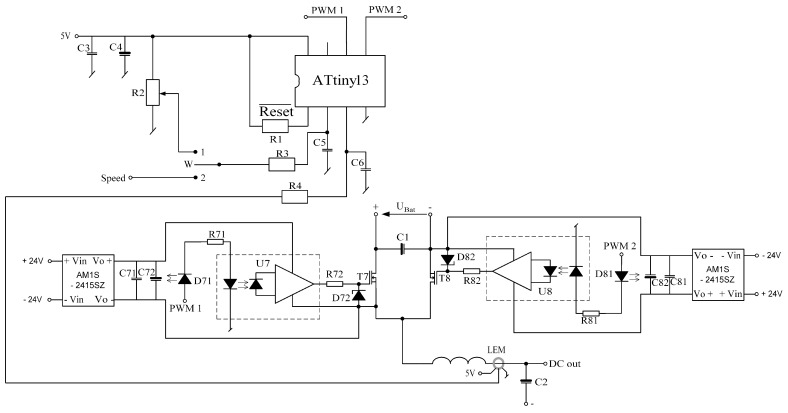
The schematic diagram of the DC/DC converter.

These transistors were selected for the motor’s power source, which consists of a battery of four series connected batteries with a rated voltage of 12 V. The selection of transistors was guided, in addition to their maximum blocking voltage, also the conduction resistance, which for this type of transistors is 8 mΩ, and their housing (dimensions). Moreover, for proper operation, galvanic isolation between the microcontroller system and the power transistors (IRF3205) is necessary. This insulation is required due to the different potentials existing on the individual windings of the motor during its operation. As elements of galvanic isolation on the stand, TLP351 optoisolators with a maximum collector-emitter voltage of 30 V were used, which is sufficient to fully open the IRF3205 transistor used. LED diodes installed on the input side of optoisolators are powered by resistors limiting the current from the output pins of the microcontroller. On the other hand, the output side of the optoisolators controlling the power transistors requires an individual power source, and the stand constructed as such sources used converters with a power of 1W type AM1S-2415SZ. These converters are 24 V powered and each converter supplies the voltage required to power each of the transistors individually. Such configuration was used both in the electronic commutator system and in the converter system used to lower the motor supply voltage. The schematic diagram of the converter is presented in Figure 12.

**Figure 13:**
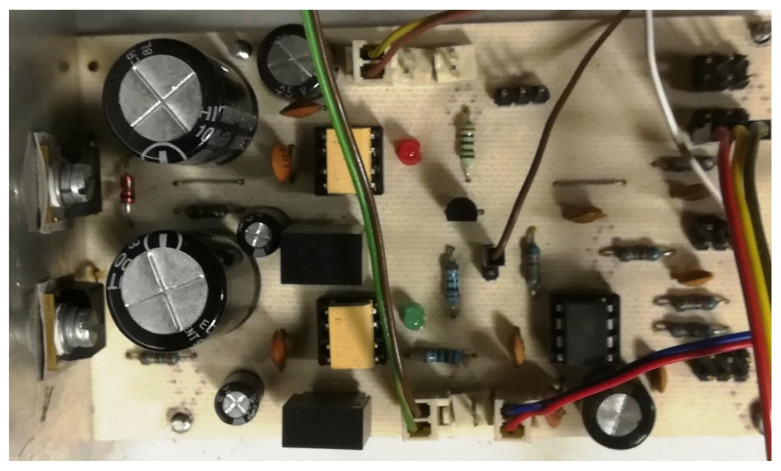
Photo of the developed DC/DC converter.

**Figure 14:**
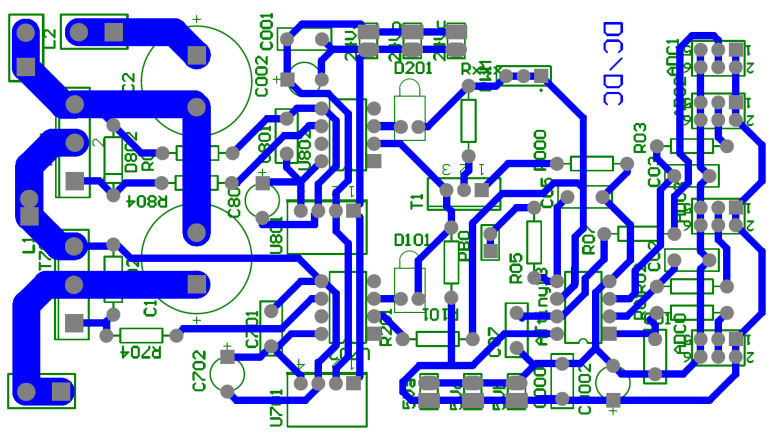
PCB board design for DC/DC converter.

The realizations of a DC/DC converter designed in the research is presented in Figure 13. PCB design made as part of the research work is presented in Figure 14.

**Figure 15:**
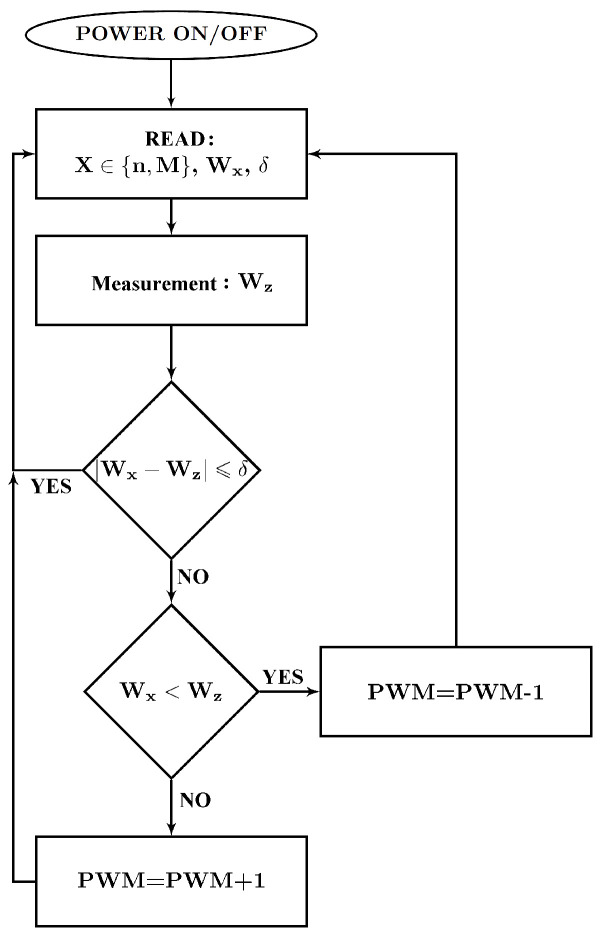
Block diagram of the converter microcontroller program to stabilize the motor spin speed.

The converter consists of two mosfet transistors, which are controlled by drivers with a signal supplied from a microcontroller. The ATtiny 13 microcontroller was used as a microcontroller for the converter. Such a microcontroller was chosen because it has analog-to-digital converters and two PWM signal channels. One of the analog-to-digital converters is supplied with a signal from a control potentiometer or an external system that sets the motor supply voltage. This signal corresponds to the desired value of the output voltage, ranging from 0 to the supply voltage supplied from the battery bank $U_{Bat}$. The converter microcontroller generates a PWM signal with such filling that the average value of the PWM signal corresponds to the set value of the motor supply voltage. The signal from the microcontroller is sent to the optical insulation system, and then the power transistors are switched on. The converter microcontroller, using a second analog-to-digital converter, also measures the current consumed from the converter, thanks to which it is possible to control the current flowing in a safe range, protecting the converter and the motor against overheating or damage due to overload. The converter is based on two identical power transistors with their control systems. These transistors work in such a way that when one is open, the other is closed. This solution allows not only to supply the engine with reduced voltage, but also to recover energy and return it to the battery during braking. During normal operation, when the motor is powered from the battery, the converter reduces the voltage supplied to the motor, while during braking, the converter increases the voltage supplied from the motor windings, which enables charging the battery with a voltage higher than the voltage obtained from the motor windings. In both cases, the converter current is measured and taken into account in the control to protect against damage. Moreover, by using the possibility of switching the source of the motor supply voltage reference thanks to the W switch, instead of setting the signal from the potentiometer, it is possible to set a signal from an external system. This solution allows for the implementation of the engine spin speed stabilization system. The block diagram of the microcontroller program realizing the spin speed stabilization is shown in Figure 15.

## 4. Measurements and Analysis of the BLDC Motor Supply Current

In order to determine the extent to which the efficiency of the drive can be improved by eliminating ripple or limiting it, measurements of the motor supply current in various operating states, i.e., for idling, and loads of various sizes, were carried out. The measurements were made in the arrangement shown in Figure 3. Later in the article, this system was called the basic system. Then, based on the obtained results, an analysis was carried out, on the basis of which the root mean square (RMS) and average (AVG) of the motor supply current were determined. The effective value is described by the formula:

(1) $$I_{RMS}=\sqrt{\frac{1}{T}\int_{t_{0}}^{T+t_{0}}i^{2}\left(t\right)dt}$$

while the average value:

(2) $$I_{AVG}=\frac{1}{T}\int_{t_{0}}^{T+t_{0}}i\left(t\right)dt$$

where, in the case of both formulas, *T* is the duration of one feed cycle and $t_{0}$ is the initial feed cycle time, and i(t) is a function of the current.

The current analysis was performed within the feed cycle. The results obtained in this way are presented in Table 1—idling and in Table 2—under load. Measurements were made for two directions of rotation.

**Figure 16:**
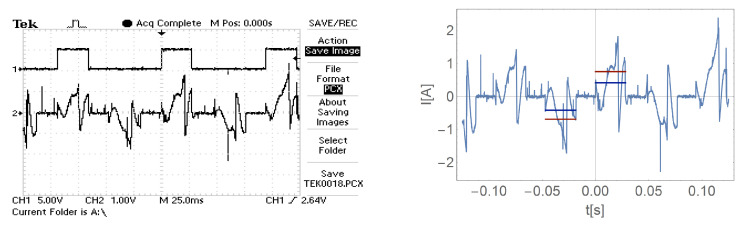
The waveform of the current of one motor phase during work with the load M0=0.

**Figure 17:**
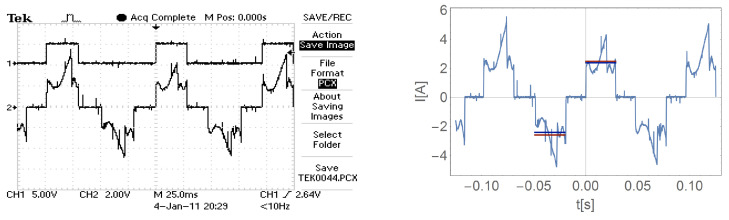
The waveform of the current of one motor phase during work with the load M1.

**Figure 18:**
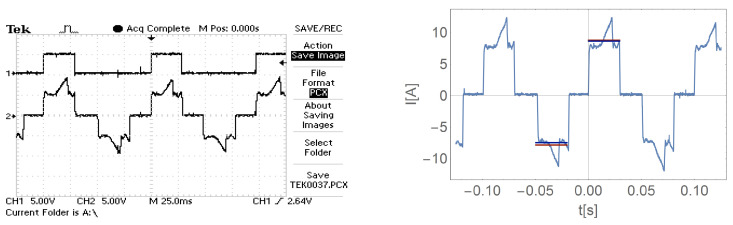
The waveform of the current of one motor phase during work with the load M2.

**Figure 19:**
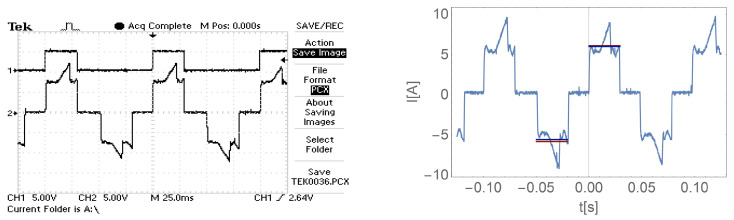
The waveform of the current of one motor phase during work with the load M3.

Figure 16, Figure 17, Figure 18 and Figure 19 show the current waveform for single cycles (current flow in both directions) of the motor supply. Each of these figures features an oscillogram, which, apart from the current waveform, also shows the duration of the power sequence. The figure on the right shows the mean value for a single measure (blue line) and the effective value of the current (red line), while the effective value is shown with a sign of the mean value for better comparison.

Figure 16, Figure 17, Figure 18 and Figure 19 and data presented in Table 1 and Table 2 show that the root mean square (RMS) value of the motor current is greater than the average value for a given feed cycle. Additionally, the analysis showed that the share of the ripple component in the motor current decreases with increasing load.

Such a result of the analysis of the motor supply current indicates the possibility of reducing losses in the motor windings by using a converter stabilizing the current in the motor winding (one cycle time), and in order to maintain the mechanical parameters of the drive, the current value should correspond to the average value within a given cycle.

## 5. Implementation of the System Reducing the Ripple of the BLDC Motor Supply Current

In order to reduce the difference between the average value and the effective value, the DC/DC converter and the program of the microcontroller controlling the DC/DC converter were modified. In order to ensure the possibility of current stabilization during one cycle of power supply to the motor, the capacitive filter located behind the DC/DC converter ($C_{Out}$) was removed from the converter. The converter has been extended with a system generating a signal proportional to the average value of the motor supply current and a system which, based on this signal, controls the power transistors of the DC/DC converter (T7 and T8). These transistors also perform the function of speed control of the drive through PWM modulation. On the other hand, the electronic commutator transistors (T1–T6) are opened only on the basis of information about the rotor position (no PWM modulation). Later in the article this system was called the modified system.

Similarly to the system without smoothing the motor supply current, measurements and analysis of the current within the feed cycle were carried out. For work at idle speed, the data are presented in Table 3, and for work under load in the Table 4.

**Figure 20:**
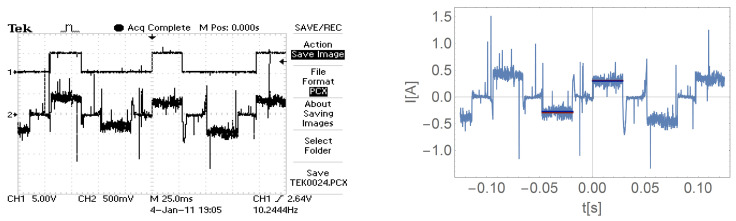
Current waveform of one motor phase in a system with current ripple limitation during load operation M=0.

**Figure 21:**
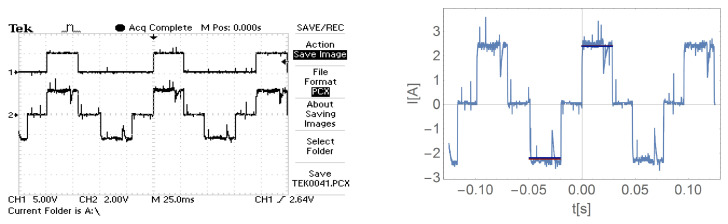
Current waveform of one motor phase in a system with current ripple limitation during load operation M1.

**Figure 22:**
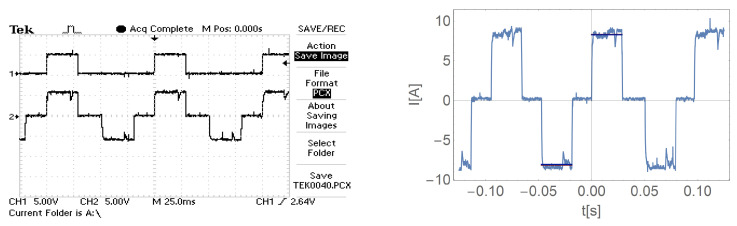
Current waveform of one motor phase in a system with current ripple limitation during load operation M2.

**Figure 23:**
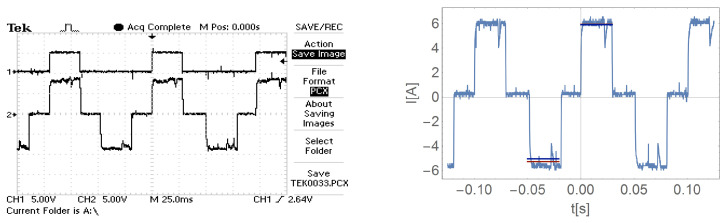
Current waveform of one motor phase in a system with current ripple limitation during load operation M3.

As for the basic system, also in the case of the modified system, Figure 20, Figure 21, Figure 22 and Figure 23 show the mean value (blue) and the effective current (red line) for a single tact, where the RMS value is shown with the sign of the mean value for a better comparison.

## 6. Comparison of the Performance of the Basic and Modified Systems

In order to verify the operation of the system reducing the ripple of the motor current (during one power cycle) in the Table 3 and Table 4, the percentage relative difference of the mean value $I_{AVG}$ was compared from the RMS value of $I_{RMS}$ supply current given by the formula

(3) $$\Delta_{I}=100\%\frac{\left|I_{RMS}-I_{AVG}\right|}{I_{AVG}}$$

and $\Delta_{P}$ parameter values were determined by the formula

(4) $$\Delta_{P}=\frac{\left|I_{RMS}^{2}-I_{AVG}^{2}\right|}{I_{AVG}^{2}}$$

which corresponds to the relative difference of the loss increment in the motor windings. Both parameters have been determined for operation without and with the motor current ripple limitation system, taking into account the positive and negative pulses.

The summary of data from Table 5 and Table 6 for $\Delta_{I}$, and from Table 7 and Table 8 for $\Delta_{P}$, shows that the current ripple limitation in one power cycle, $\Delta_{I}$ parameter significantly reduces under certain operating conditions (low load), and thus the losses resulting from the current flow ($\Delta_{P}$ parameter).

**Figure 24:**
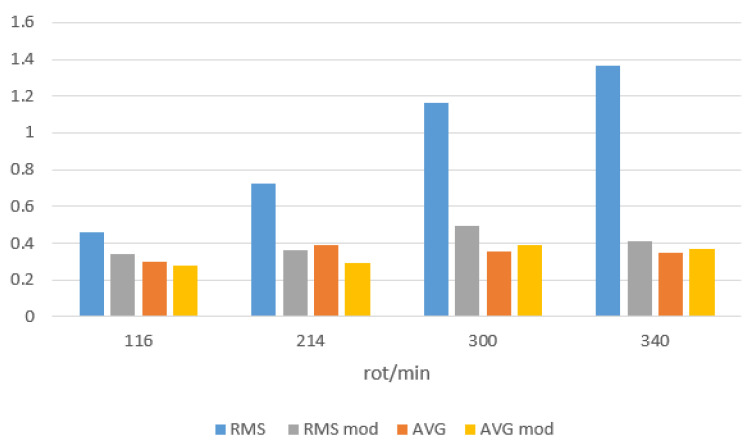
Comparison of the mean and effective values for the basic system and the modified system for idling with different spin speeds.

Figure 24 shows a comparison of averaged values of mean and averaged RMS values of the motor winding current for idle operation in both directions of rotation and for one pulse. The results are presented for different engine spin speeds. It can be read from the graph that in the modified system the effective value significantly decreases in relation to the basic system at a comparable mean value. What should be interpreted as a change in losses resulting from the current flow in the motor windings.

## 7. Conclusions

The analysis of the BLDC motor tests carried out showed that the mean current value differs significantly from the effective value during one power cycle. This observation was the basis for an attempt to reduce losses resulting from the current flow in the motor windings by reducing the current pulsation during one power cycle. In order to achieve this goal, it was decided to modify the DC/DC converter system in such a way as to stabilize the current flowing in the motor windings. In the modified circuit, the output capacitance $C_{Out}$ was removed from the DC/DC converter and the program in the microprocessor controlling the DC/DC converter was changed.

The presented results of the analysis show that the elimination of the motor current ripple component (during one feed cycle) significantly reduces the RMS value of the motor supply current during idling and part load operation. As the load increases, the system is characterized by the fact that the difference between the effective value and the average current value decreases (during one feed cycle). During high load operation, the difference between the RMS value and the average current value is so small that an attempt to limit the ripple of the motor current does not bring any significant benefits. The use of the motor current ripple component elimination system is justified in the case of drives with a significant idle time. In the case of other systems, i.e., those for which the time of no-load or light-load operation is negligible in the work cycle, it is not necessary to eliminate the ripple component of the motor supply current (during one cycle), due to its small share.

## Figures and Tables

**Table 1:** Summary of the average (AVG) and root mean square (RMS) of the current in single engine feed cycles, during right and left spinning at different idling speeds.

*rpm* [min^−1^]	Spin Left				Spin Right			
	Positive Pulse		Negative Pulse		Positive Pulse		Negative Pulse	
	RMS [A]	AVG [A]	RMS [A]	AVG [A]	RMS [A]	AVG [A]	RMS [A]	AVG [A]
116	0.55	0.38	0.40	−0.22	0.37	0.25	0.52	−0.34
214	0.76	0.35	0.71	−0.37	0.76	0.42	0.68	−0.41
300	1.14	0.38	1.34	−0.30	1.16	0.52	1.00	−0.21
340	1.39	0.40	1.42	−0.27	1.22	0.32	1.44	−0.41
400	1.47	0.33	1.60	−0.25	1.45	0.32	1.70	−0.43
500	1.45	0.39	1.55	−0.28	1.65	0.37	1.92	−0.52
540	1.53	0.42	1.44	−0.38	1.93	0.64	1.62	−0.53

**Table 2:** Summary of the average (AVG) and root mean square (RMS) of the current in single power cycles of the motor, during right and left spinning with the speed rpm=200 min${}^{-1}$ and with different loads (M0<M1<M2<M3).

Load	Spin Left				Spin Right			
	Positive Pulse		Negative Pulse		Positive Pulse		Negative Pulse	
	RMS [A]	AVG [A]	RMS [A]	AVG [A]	RMS [A]	AVG [A]	RMS [A]	AVG [A]
M0	0.76	0.35	0.71	−0.37	0.76	0.42	0.68	−0.41
M1	2.53	2.47	2.38	−2.17	2.50	2.40	2.58	−2.40
M2	5.88	5.81	5.37	−5.25	6.09	5.97	5.84	−5.60
M3	8.23	8.17	7.68	−7.53	8.80	8.67	7.80	−7.43

**Table 3:** Summary, for the modified system, of the average (AVG) and root mean square (RMS) of the current in single engine feed cycles, during right rotation at different idling speeds.

*rpm* [min−1]	Spin Left				Spin Right			
	Positive Pulse		Negative Pulse		Positive Pulse		Negative Pulse	
	RMS [A]	AVG [A]	RMS [A]	AVG [A]	RMS [A]	AVG [A]	RMS [A]	AVG [A]
116	0.42	0.36	0.28	−0.18	0.23	0.18	0.43	−0.39
214	0.37	0.35	0.48	−0.26	0.31	0.30	0.29	−0.27
300	0.43	0.42	0.58	−0.28	0.49	0.46	0.48	−0.40
340	0.36	0.35	0.43	−0.35	0.40	0.36	0.44	−0.42

**Table 4:** Summary, for the modified system, the average (AVG) and root mean square (RMS) of the current in single engine power cycles, during clockwise rotation with the speed rpm = 200 min${}^{-1}$ and with different loads (M0<M1<M2<M3).

Load	Spin Left				Spin Right			
	Positive Pulse		Negative Pulse		Positive Pulse		Negative Pulse	
	RMS [A]	AVG [A]	RMS [A]	AVG [A]	RMS [A]	AVG [A]	RMS [A]	AVG [A]
M0	0.31	0.30	0.29	−0.27	0.37	0.35	0.39	−0.35
M1	2.41	2.39	2.23	−2.19	2.40	2.39	2.28	−2.14
M2	5.91	5.91	5.27	−5.04	5.87	5.86	5.24	−5.15
M3	8.32	8.28	8.12	−8.07	8.40	8.38	7.53	−7.22

**Table 5:** Comparison of the value of the $\Delta_{I}$ parameter describing the difference between $I_{RMS}$$I_{AVG}$ for idling with different speeds for the basic system—BS and the modified system (modified system)—MS.

*rpm* [min^−1^]	Spin Left				Spin Right			
	Positive Pulse		Negative Pulse		Positive Pulse		Negative Pulse	
	$\Delta_{I_{\mathrm{BS}}}$ [%]	$\Delta_{I_{\mathrm{MS}}}$ [%]	$\Delta_{I_{\mathrm{BS}}}$ [%]	$\Delta_{I_{\mathrm{MS}}}$ [%]	$\Delta_{I_{\mathrm{BS}}}$ [%]	$\Delta_{I_{\mathrm{MS}}}$ [%]	$\Delta_{I_{\mathrm{BS}}}$ [%]	$\Delta_{I_{\mathrm{MS}}}$ [%]
116	30.9	14.3	45.0	35.7	32.4	21.7	34.6	9.3
214	53.9	5.4	47.9	45.8	44.7	3.2	39.7	6.9
300	66.6	2.3	77.6	51.7	55.2	6.1	79.0	16.7
340	71.2	2.8	81.0	18.6	73.8	10.0	71.5	4.5

**Table 6:** Comparison of $\Delta_{I}$ parameter value describing the difference between $I_{RMS}$$I_{AVG}$ for rpm=200 min${}^{-1}$ and with different engine loads (M=0<M1<M2<M3 ) for basic system—BS and modified system—MS.

Load	Spin Left				Spin Right			
	Positive Pulse		Negative Pulse		Positive Pulse		Negative Pulse	
	$\Delta_{I_{\mathrm{BS}}}$ [%]	$\Delta_{I_{\mathrm{MS}}}$ [%]	$\Delta_{I_{\mathrm{BS}}}$ [%]	$\Delta_{I_{\mathrm{MS}}}$ [%]	$\Delta_{I_{\mathrm{BS}}}$ [%]	$\Delta_{I_{\mathrm{MS}}}$ [%]	$\Delta_{I_{\mathrm{BS}}}$ [%]	$\Delta_{I_{\mathrm{MS}}}$ [%]
*M*0	53.9	3.3	47.9	6.9	44.7	5.4	39.7	11.43
*M*1	2.4	0.8	8.8	1.8	4.0	0.4	7.0	6.5
*M*2	1.1	0	2.2	4.5	2.0	0.2	4.3	1.7
*M*3	0.7	0.5	1.9	0.6	1.4	0.2	4.7	4.1

**Table 7:** Comparison of the value of the Δp parameter describing the relative difference of the power loss increment for idling with different speeds during spinning in both directions for the basic system—BS—and modified system—MS.

*rpm* [min^−1^]	Spin Left				Spin Right			
	Positive Pulse		Negative Pulse		Positive Pulse		Negative Pulse	
	$\Delta_{p_{\mathrm{BS}}}$	$\Delta_{p_{\mathrm{MS}}}$	$\Delta_{p_{\mathrm{BS}}}$	$\Delta_{p_{\mathrm{MS}}}$	$\Delta_{p_{\mathrm{BS}}}$	$\Delta_{p_{\mathrm{MS}}}$	$\Delta_{p_{\mathrm{BS}}}$	$\Delta_{p_{\mathrm{MS}}}$
116	1.09	0.36	2.31	1.42	1.19	0.63	1.34	0.22
214	3.71	0.12	2.68	2.41	2.27	0.07	1.75	0.15
300	8.00	0.05	18.95	3.29	3.98	0.13	21.68	0.44
340	11.08	0.06	26.66	0.51	13.54	0.23	11.34	0.10

**Table 8:** Comparison of the value of the Δp parameter describing the relative difference in the loss increment for right centrifugation with the speed rpm=200 min${}^{-1}$ and with different engine loads (M=0<M1<M2<M3) for the basic system—BS—and modified system—MS.

Load	Spin Left				Spin Right			
	Positive Pulse		Negative Pulse		Positive Pulse		Negative Pulse	
	$\Delta_{p_{\mathrm{BS}}}$	$\Delta_{p_{\mathrm{MS}}}$	$\Delta_{p_{\mathrm{BS}}}$	$\Delta_{p_{\mathrm{MS}}}$	$\Delta_{p_{\mathrm{BS}}}$	$\Delta_{p_{\mathrm{MS}}}$	$\Delta_{p_{\mathrm{BS}}}$	$\Delta_{p_{\mathrm{MS}}}$
*M*0	3.72	0.07	2.68	0.15	2.27	0.12	1.75	0.24
*M*1	0.05	0.02	0.20	0.37	0.09	0.01	0.16	0.14
*M*2	0.02	0.00	0.05	0.09	0.04	0.00	0.09	0.04
*M*3	0.01	0.01	0.04	0.01	0.03	0.00	0.10	0.09

## References

[1] Sakunthala S., Kiranmayi R., Mandadi P.N. (2017). A study on industrial motor drives: Comparison and applications of PMSM and BLDC motor drives. Proceedings of the 2017 International Conference on Energy, Communication, Data Analytics and Soft Computing (ICECDS).

[2] Zhou X., Chen X., Lu M., Zeng F. (2017). Rapid self-compensation method of commutation phase error for low-inductance BLDC motor. IEEE Trans. Ind. Inform..

[3] Mohammad A., Khan M.Z.R. (2015). BLDC motor controller for Regenerative Braking. Proceedings of the 2015 International Conference on Electrical Engineering and Information Communication Technology (ICEEICT).

[4] Jayetileke H., de Mel W., Ratnayake H. (2017). Modelling and simulation analysis of the genetic-fuzzy controller for speed regulation of a Sensored BLDC motor using Matlab/Simulink. Proceedings of the 2017 IEEE International Conference on Industrial and Information Systems (ICIIS).

[5] Masmoudi M., El Badsi B., Masmoudi A. (2013). DTC of B4-inverter-fed BLDC motor drives with reduced torque ripple during sector-to-sector commutations. IEEE Trans. Power Electron..

[6] Usman A., Rajpurohit B.S. (2016). Speed control of a BLDC motor using fuzzy logic controller. Proceedings of the 2016 IEEE 1st International Conference on Power Electronics, Intelligent Control and Energy Systems (ICPEICES).

[7] Errabelli R.R., Mutschler P. (2011). Fault-tolerant voltage source inverter for permanent magnet drives. IEEE Trans. Power Electron..

[8] Wang X., Fan W., Li X., Wang L. (2019). Weak degradation characteristics analysis of UAV motors based on laplacian eigenmaps and variational mode decomposition. Sensors.

[9] Yedamale P. (2003). Brushless DC (BLDC) Motor Fundamentals.

[10] Zhao L., Zhang X., Ji J. A torque control strategy of brushless direct current motor with current observer. Proceedings of the 2015 IEEE International Conference on Mechatronics and Automation (ICMA).

[11] Shao J. (2006). An improved microcontroller-based sensorless brushless DC (BLDC) motor drive for automotive applications. IEEE Trans. Ind. Appl..

[12] Morimoto S., Kawamoto K., Sanada M., Takeda Y. (2002). Sensorless control strategy for salient-pole PMSM based on extended EMF in rotating reference frame. IEEE Trans. Ind. Appl..

[13] Zhu H., Xiao X., Li Y. (2011). Torque ripple reduction of the torque predictive control scheme for permanent-magnet synchronous motors. IEEE Trans. Ind. Electron..

[14] Liu H., Li S. (2011). Speed control for PMSM servo system using predictive functional control and extended state observer. IEEE Trans. Ind. Electron..

[15] Liu Y., Zhu Z., Howe D. (2005). Direct torque control of brushless DC drives with reduced torque ripple. IEEE Trans. Ind. Appl..

[16] Aghili F. (2010). Fault-tolerant torque control of BLDC motors. IEEE Trans. Power Electron..

[17] Park S.J., Park H.W., Lee M.H., Harashima F. (2000). A new approach for minimum-torque-ripple maximum-efficiency control of BLDC motor. IEEE Trans. Ind. Electron..

[18] Nasri M., Nezamabadi-Pour H., Maghfoori M. (2007). A PSO-based optimum design of PID controller for a linear brushless DC motor. World Acad. Sci. Eng. Technol..

[19] Jeon Y., Mok H., Choe G., Kim D., Ryu J. (2000). A new simulation model of BLDC motor with real back EMF waveform. Proceedings of the COMPEL 2000 7th Workshop on Computers in Power Electronics. Proceedings (Cat. No. 00TH8535).

[20] Jang G.H., Kim M. (2006). Optimal commutation of a BLDC motor by utilizing the symmetric terminal voltage. IEEE Trans. Magn..

[21] Ozturk S.B., Toliyat H.A. (2007). Direct torque control of brushless dc motor with non-sinusoidal back-EMF. Proceedings of the 2007 IEEE International Electric Machines & Drives Conference.

[22] Singh S., Verma K.K.S.D.K., Singh J., Tiwari N. (2018). A Review on Control of a Brushless DC Motor Drive. Int. J. Future Revolution Comput. Sci. Commun. Eng..

[23] Fang J., Zhou X., Liu G. (2012). Precise accelerated torque control for small inductance brushless DC motor. IEEE Trans. Power Electron..

[24] Niapour S.K.M., Danyali S., Sharifian M., Feyzi M. (2011). Brushless DC motor drives supplied by PV power system based on Z-source inverter and FL-IC MPPT controller. Energy Convers. Manag..

[25] Lu H., Zhang L., Qu W. (2008). A new torque control method for torque ripple minimization of BLDC motors with un-ideal back EMF. IEEE Trans. Power Electron..

[26] Arun Prasad K., Usha N. (2019). Design and FPGA Implementation of Non-Linear Intelligent Control for Special Electric Drives. Ph.D. Thesis.

[27] Tarmizi Y.A., Jidin A., Karim K.A., Sutikno T. (2019). A simple constant switching frequency of direct torque control of brushless DC motor. Int. J. Power Electron. Drive Syst..

[28] Muralidhar J., Aranasi P. (2014). Torque ripple minimization & closed loop speed control of BLDC motor with hysteresis current controller. Proceedings of the 2014 2nd International Conference on Devices, Circuits and Systems (ICDCS).

[29] Ransara H.S., Madawala U.K. (2015). A torque ripple compensation technique for a low-cost brushless DC motor drive. IEEE Trans. Ind. Electron..

[30] Yen S.H., Tang P.C., Lin Y.C., Lin C.Y. (2019). A Sensorless and Low-Gain Brushless DC Motor Controller Using a Simplified Dynamic Force Compensator for Robot Arm Application. Sensors.

